# Circular RNA hsa_circ_0000117 accelerates the proliferation and invasion of gastric cancer cells by regulating the microRNA-337-3p/signal transducer and activator of transcription 3 axis

**DOI:** 10.1080/21655979.2021.1918992

**Published:** 2021-04-25

**Authors:** Qin Gao, Qilin Liu, Hongfei Chen

**Affiliations:** a Department of Anesthesia, Affiliated Hospital of North Sichuan Medical College, Nanchong, Sichuan, China; b Department of Anesthesia, Shanghai East Hospital, Tongji University School of Medicine, Shanghai, China

**Keywords:** Gastric cancer, hsa_circ_0000117, miR-337-3p, STAT3

## Abstract

Circular RNA hsa_circ_0000117 is reportedly increased in Gastric cancer (GC), however, its role is unexplored. Hsa_circ_0000117 expression and function in GC was investigated using standard cell phenotypic and expression assays. Pull-down and luciferase reporter assays also elucidated hsa_circ_0000117 mechanisms. In the present study, we observed increased hsa_circ_0000117 and signal transducer and activator of transcription 3 (STAT3) expression, while microRNA-337-3p (miR-337-3p) was decreased in GC cells. Depleted hsa_circ_0000117 decreased GC proliferation and invasion. Hsa_circ_0000117 was also identified as a miR-337-3p sponge. Also, STAT3 was identified as a miR-337-3p target. Similarly, rescue assays indicated STAT3 overexpression (or miR-337-3p inhibition) reversed hsa_circ_0000117 effects in GC progression. Thus, our data suggested hsa_circ_0000117 exhibited oncogene properties in combination with the hsa_circ_0000117/miR-337-3p/STAT3 axis in GC, potentially providing a new therapeutic target for GC.

**Abbreviations** GC: gastric cancer; STAT3: Signal transducer and activator of transcription 3; circRNA: Circular RNA; miRNA: microRNA; DMEM: Dulbecco’s modified Eagle’s medium; FBS: fetal bovine serum; PVDF: polyvinylidene fluoride; CCK-8: Cell counting kit-8; qRT-PCR: Quantitative real-time PCR; SDS-PAGE: sodium dodecyl sulfate polyacrylamide gel electrophoresis; TNM: TNM Classification of Malignant Tumors; mTOR: mechanistic target of rapamycin; ANOVA: one-way analysis of variance

Gastric cancer (GC) is a common cancer, and is the third most common cause of cancer death [1]. In 2018, GC culminated in 783000 worldwide deaths and was diagnosed in approximately 1,000,000 individuals [2]. Several factors are implicated in GC etiology, including, Helicobacter pylori infection, eating behaviors, smoking, and being overweight [3]. In recent years, while diagnostic progress and GC standardized treatments have occurred, the prognosis for patients with advanced GC stages remains unsatisfactory [4,5]. Therefore, the molecular mechanisms behind GC tumor progression must be investigated to improve GC treatments.

Circular RNA (circRNA) is a non-coding RNA, and is more stable than linear RNA [6]. Studies have shown circRNAs are dynamically expressed in human cancers and regulate gene expression, suggesting a close association with tumor occurrence and invasiveness [7,8]. It was reported circMTO1 suppressed cancer cell properties by targeting Wnt/β-catenin signaling in colorectal cancer [9]. Also, circPRRC2A promoted cancer cell characteristics via epithelial-mesenchymal transition (EMT), and increased TRPM3 levels in renal cell carcinoma [10]. Similarly, circRNA hsa_circ_0008285 increased cervical cancer cell properties by activating SOX4, and inhibiting miR-211-5p [11]. Equally, hsa_circ_0000117 was highly abundant in GC [12]. However, hsa_circ_0000117 roles and its underlying mechanism in GC are still unclear.

MicroRNAs (miRNAs) are another crucial class of non-coding RNAs with approximately 21–23 nucleotides in length [13]. Recently, miR-337-3p has been established to be a tumor inhibitor in various human cancers, including GC. For instance, loss of miR-337-3p expression in GC was correlated with lymph node metastasis [14]. Also, miR-337-3p suppressed GC cells proliferation and invasion of GC cells through repressing MZF1-facilitated expression of MMP14 [15]. Similarly, miR-337-3p reduced GC cells growth and invasion through regulating ARHGAP10 [16]. Increasing studies have demonstrated that circRNAs could function as direct sponges for miRNAs to play their essential biological functions in eukaryotic cells [17,18]. However, the relationship between hsa_circ_0000117 and miR-337-3p has not been studied.

Here, we explored the biological functions and molecular mechanisms of hsa_circ_0000117. We identified that the hsa_circ_0000117/miR-337-3p/STAT3 feedback loop enhanced GC cell proliferation and invasion, suggesting a new treatment strategy for GC.

## Methods and materials

### Patient tissues

Thirty-nine (39) tumor specimen pairs and matched normal samples were obtained from the Affiliated Hospital of North Sichuan Medical College. GC surgery is provided at this facility. All patients were informed and signed consent forms. The research was approved by the local Ethics Committee. Participants received no preoperative treatment prior to study commencement, and were diagnosed with GC by two independent pathologists. Tissue specimens were stored at −80°C.

### Cell culture

The human immortalized gastric epithelial cell line (GES-1) and five GC cell lines (AGS, HGC-27, SGC7901, BGC823, and SNU-1) were provided by the Chinese Academy of Sciences (Shanghai, China). Cells were grown in DMEM (Invitrogen, CA, USA) containing 10% FBS (Gibco, CA, USA) in 5% CO_2_ at 37°C.

Specific small interfering (si)RNAs against hsa_circ_0000117 (si-circ_0000117) or STAT3 (si-STAT3) and their corresponding NC’s (si-NC) were purchased from Genechem (Shanghai, China). The miR-337-3p mimic, miR-337-3p inhibitor, and corresponding controls were purchased from Geneseed (Shanghai, China). Reagents were transfected into GC cells using lipofectamine 3000 (Invitrogen).

### Quantitative real-time PCR

Total RNA was isolated from tissue or cells using TRIzol® (Invitrogen) and cDNA synthesized using the miScript Reverse Transcription Kit (Qiagen, Germany). After reverse transcription, qPCR was conducted using the miScript SYBR Green PCR Kit (Qiagen) to quantify miR-337-3p levels with respect to U6 small nuclear RNA. To quantify hsa_circ_0000117 and STAT3, cDNAs were generated by the PrimeScript RT-reagent kit (Takara, Japan), and levels standardized to GAPDH expression. Data were processed using the 2^−ΔΔCt^ approach.

### Cell counting assay

Cells were plated in 96-well plates (2000 cells/well) and grown for 0, 24, 48, 72, 96 h. CCK-8 kit-8 (Dojindo, Japan) was used according to manufacturer’s instructions. A spectrophotometer reader (Molecular Devices, CA, USA) recorded absorbance at 450 nm.

### Transwell assay

2 × 10^5^ cells were cultured in an upper transwell chamber containing Matrigel (BD Biosciences, NJ, USA) in 200 μl medium lacking serum. The lower chamber contained 600 μl complete medium. After a 48 h incubation, the upper chamber was removed, and 4% paraformaldehyde and 0.5% crystal violet sequentially added to fix and stain cells, respectively, in the lower chamber. A light microscope (Nikon, Japan) was used to count cells.

### Dual-luciferase reporter assay

To show miR-337-3p targeted hsa_circ_0000117 and the targeting relationship of miR-337-3p and STAT3, wild-type or mutated sequences of hsa_circ_0000117 and STAT3 were sub-cloned into pmirGLO reporter plasmids (Promega, Wisconsin, USA). PmirGLO-hsa_circ_0000117-Wt and PmirGLO-hsa_circ_0000117-Mut (or pmirGLO-STAT3-Wt and pmirGLO-STAT3-Mut) reporters were co-transfected with miR-337-3p or NC mimics into cells. After 48 h, cells were assessed for luciferase activity using the dual-luciferase reporter kit (Promega) and a spectrophotometer.

### Western blotting

The extracted proteins were measure by BCA Protein Assay Kit (Thermo, USA), separated by SDS-PAGE, transferred to PVDF membrane, and blocked in 5% fat-free milk for 1 h. Then, the membranes were incubated with primary antibodies against STAT3 (Santa Cruz, CA, USA). Secondary antibody was added and incubated for 2 h at 37℃. Finally, the immunofluorescence of protein was visualized using an ECL detection system.

### Pull-down assay

Pull-down assay was used as previous study [10]. Briefly, hsa_circ_0000117 probe was used for incubation with M-280 Streptavidin magnetic beads for 2 h at room temperature to generate probe-coated beads (Invitrogen). Cell lysate with hsa_circ_0000117 probe or oligo probe was incubated for a night at 4℃. Then, the RNA mix bound to the beads was eluted and extracted with a RNeasy Mini Kit for qRT–PCR (Qiagen).

### Statistical analysis

SPSS 18.0 was used for statistical analysis. Data were represented as the mean ± SD. Student’s t-test compared two groups, and ANOVA demonstrated differences between more than two groups. P < 0.05 indicated statistical significance.

## Results

### Hsa_circ_0000117 is upregulated in GC

**Figure 1.**
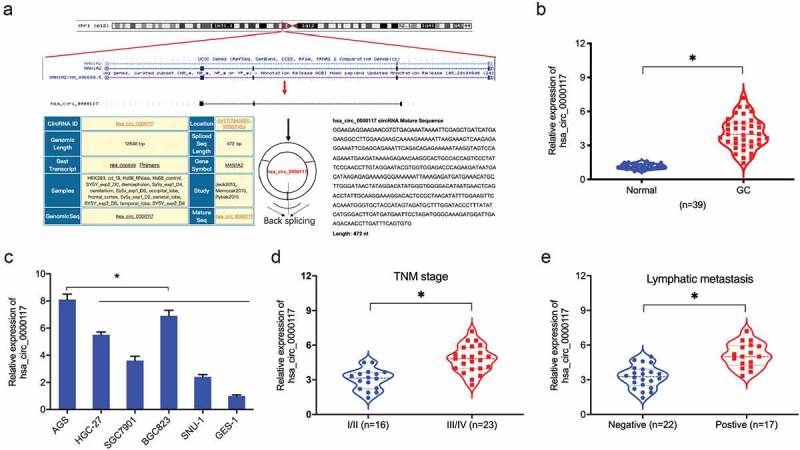
Hsa_circ_0000117 levels in GC. (a) Hsa_circ_0000117 schematic. (b) Hsa_circ_0000117 levels in GC tissue. (c) Hsa_circ_0000117 levels in GC cells. (d) Hsa_circ_0000117 expression is linked to TNM stage in GC patients. (e) Hsa_circ_0000117 expression is linked to lymphatic metastasis in GC patients. *P < 0.05

Previously, circRNA hsa_circ_0000117 expression was shown to be increased in GC [12]. However, the roles of hsa_circ_0000117 is still unclear in GC progression. In the present study, our expression data indicated hsa_circ_0000117 levels were indeed elevated in GC tissue and cell lines (Figure 1(a–c)). Additionally, correlation analyses showed this expression was positively correlated with advanced TNM stage, and lymphatic metastasis in GC patients (Figure 1(d–e)). Thus, hsa_circ_0000117 appeared to play a critical role in GC development.

### Hsa_circ_0000117 silencing reduces GC proliferation and invasion

**Figure 2.**
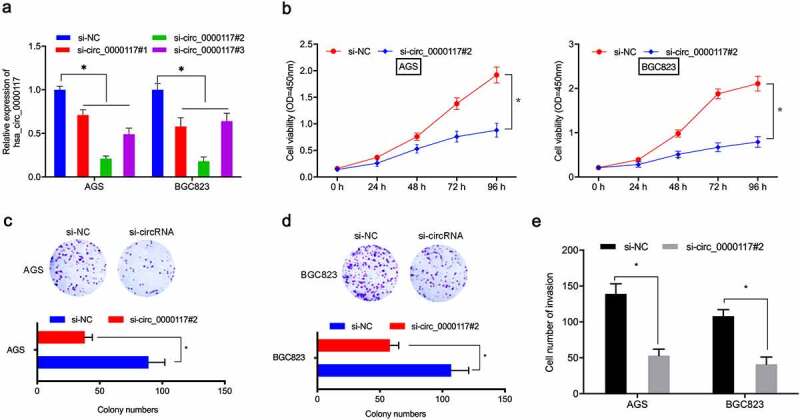
Hsa_circ_0000117 silencing suppresses GC cell proliferation and invasion. (a) Quantitative RT-PCR verified the interference efficiency of si-hsa_circ_0000117 in GC cells. (b–d) Phenotypic growth assays assessing the silencing effects of hsa_circ_0000117 on AGS and BGC823 proliferation. (e) Transwell assays assessed the effects of si-circ_0000117 on AGS and BGC823 invasion. *P < 0.05

To assess hsa_circ_0000117 mechanisms in GC tumorigenesis, AGS and BGC823 cells were transfected with si-circ_0000117 to silence expression (Figure 2(a)). CCK-8 and colony formation studies indicated this silencing decreased *in vitro* GC proliferation and colony formation (Figure 2(b–d)). Transwell assay data also indicated hsa_circ_0000117 knockdown impeded GC cell invasion (Figure 2(e)). Combined, hsa_circ_0000117 silencing appeared to suppress key GC phenotypes.

### Hsa_circ_0000117 is a sponge for miR-337-3p

**Figure 3.**
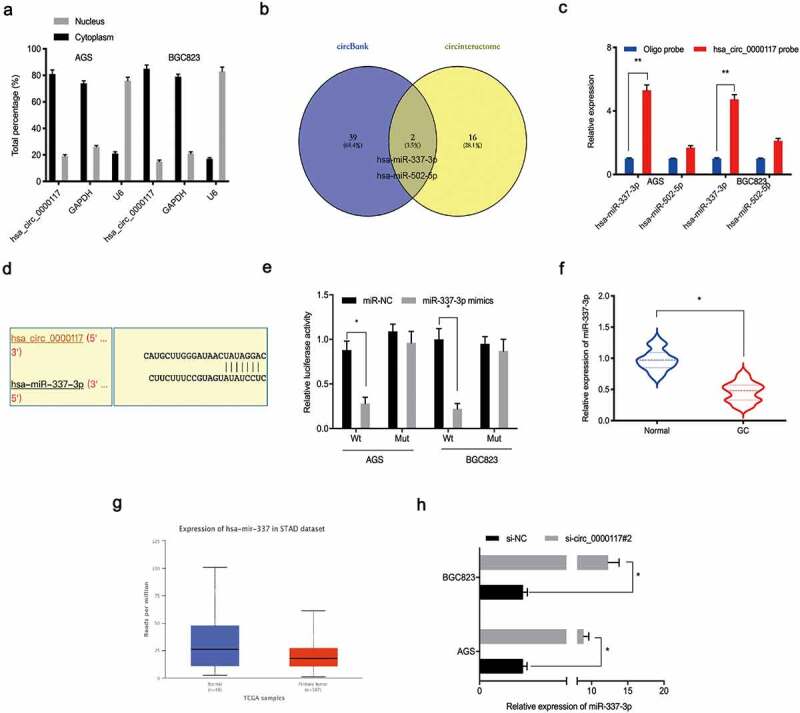
Hsa_circ_0000117 is a miR-337-3p sponge. (a) Hsa_circ_0000117 distribution in GC cells. (b) Target miRNAs of hsa_circ_0000117 were predicted using circInteractome and circBank databases. (c) MiR-337-3p and miR-502-5p binding to hsa_circ_0000117 in GC cells by pull-down assay. (d, e) The miR-337-3p/hsa_circ_0000117 relationship was characterized using a dual-luciferase reporter system. (f, g) MiR-337-3p levels in GC tissue. (h) Hsa_circ_0000117 silencing elevated miR-337-3p expression in GC cells. *P < 0.05

Increasing evidence suggests circRNAs are sponges for miRNAs, affecting downstream activities [17,18]. Therefore, we explored underlying hsa_circ_0000117 regulatory mechanisms in GC. A nuclear/cytoplasmic fractionation assay showed hsa_circ_0000117 was cytoplasmic in GC cells (Figure 3(a)). Next, potential miRNAs inactivated by hsa_circ_0000117, were investigated using circInteractome and circBank databases. These data indicated hsa-miR-337-3p and hsa-miR-502-5p exhibited complementary base pairing with hsa_circ_0000117 (Figure 3(b)). Next, RNA pull-down assays showed miR-337-3p was pulled down by biotin-hsa_circ_0000117 in AGS and BGC823 cells (Figure 3(c)). Our reporter system revealed miR-337-3p overexpression reduced luciferase intensity of the hsa_circ_0000117-WT group in GC cells (Figure 3(d,e)).

Next, we examined miR-337-3p expression in GC tissue. Our qRT-PCR data indicated this expression was downregulated in GC tissue, and corroborated by the TCGA database (figure 3(f–g)). Moreover, hsa_circ_0000117 silencing elevated miR-337-3p expression in GC cells (Figure 3(h)). Together, hsa_circ_0000117 appeared to act as a sponge for miR-337-3p in GC cells.

### STAT3 is a downstream target of miR-337-3p

**Figure 4.**
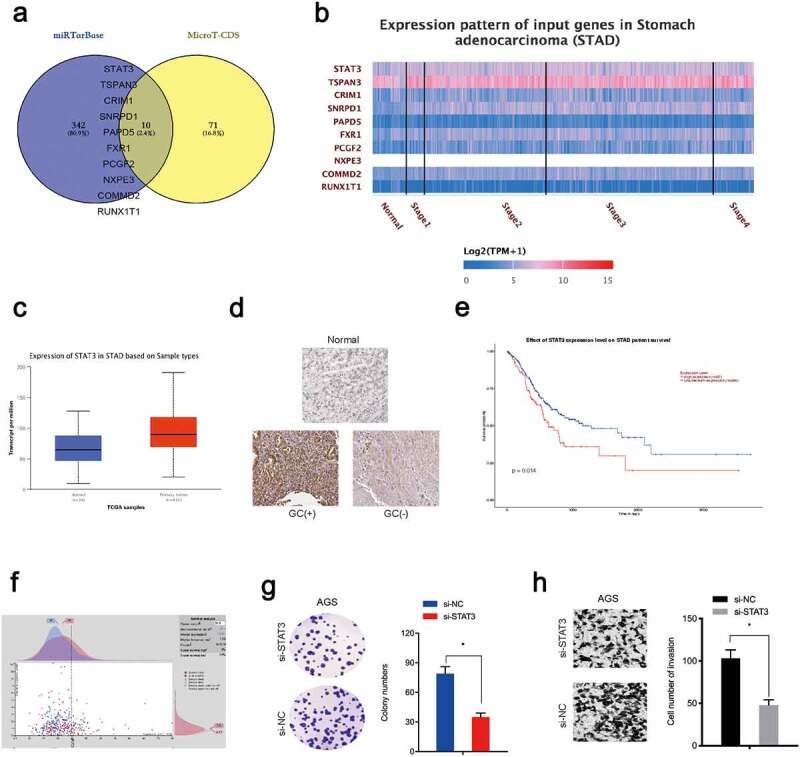
STAT3 is a target of miR-337-3p in GC cells. (a) Target genes for miR-337-3p were investigated using mirTarBase and MicroT-CDS databases. (b, c) Target gene expression using the TCGA database. (d) Immunohistochemistry showing STAT3 expression in GC tissues. (e, f) Kaplan-Meier analysis showing elevated STAT3 expression was associated with poor overall survival in GC patients. (g, h) STAT3 inhibition decreased AGS colony formation and invasion. *P < 0.05

To investigate miR-337-3p target genes, mirTarBase and MicroT-CDS databases were searched. Intersection analyses identified 10 potential genes (Figure 4(a)) which were explored using the TCGA database, from which STAT3 was chosen (Figure 4(b–c)). Immunohistochemistry data revealed STAT3 levels were increased in GC tissue (Figure 4(d)). Kaplan-Meier analysis revealed elevated STAT3 expression was linked with poor overall survival in GC patients (Figure 4(e–f)). Moreover, function assays showed STAT3 inhibition decreased *in vitro* colony formation and invasion capacities in AGS cells (Figure 4(g–h)).

**Figure 5.**
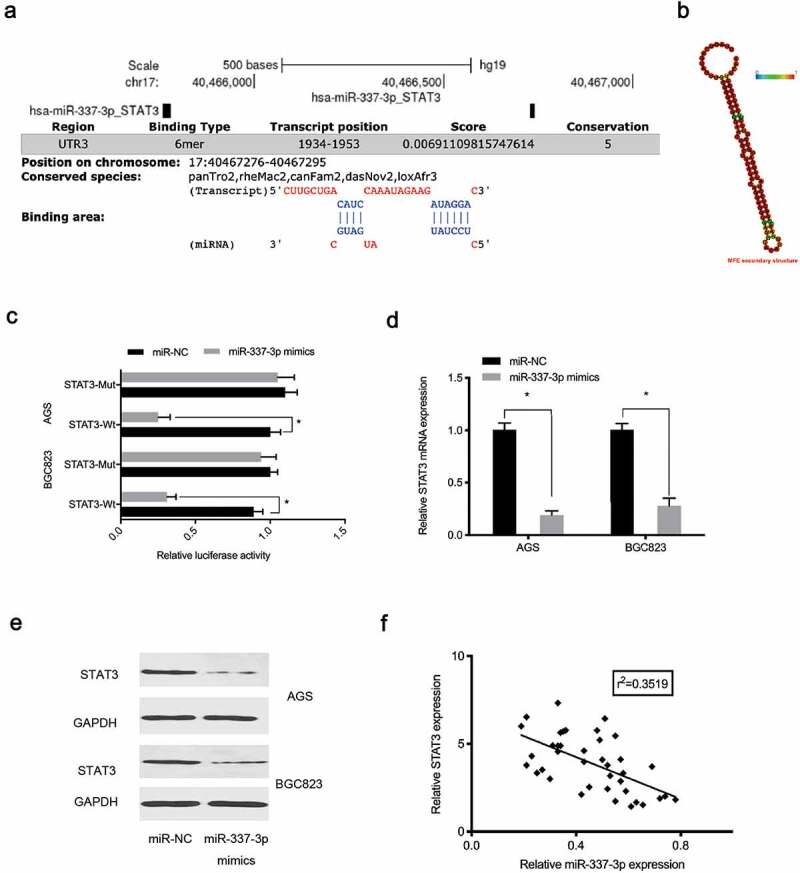
MiR-337-3p targets STAT3 in GC. (a) STAT3 binding sites for miR-337-3p were predicted using online software. (b) MiR-337-3p secondary structure. (c) Targeting interactions between STAT3 and miR-337-3p were analyzed using the dual-luciferase reporter assay. (d, e) MiR-337-3p mimics reduced STAT3 levels in AGS and BGC823 cells. (f) The association between miR-337-3p and STAT3 in GC tissue. *P < 0.05

Next, potential binding sites between miR-337-3p and the STAT3 3ʹ-untranslated region (UTR) were investigated (Figure 5(a–b)). Our reporter system indicated that forced overexpression of miR-337-3p reduced the luciferase activity of the STAT3 3ʹ-UTR-WT group, while no effects were observed for the STAT3 3ʹ-UTR-MUT group (Figure 5(c)). Expression data indicated miR-337-3p mimics decreased STAT3 levels in AGS and BGC823 cells (Figure 5(d-e)), while, correlation analysis showed miR-337-3p expression was negatively correlated with STAT3 levels in GC tissue (Figure 5(f)), suggesting miR-337-3p targeted STAT3 in GC cells.

### Hsa_circ_0000117 enhances GC malignancy via the miR-337-3p/STAT3 axis

**Figure 6.**
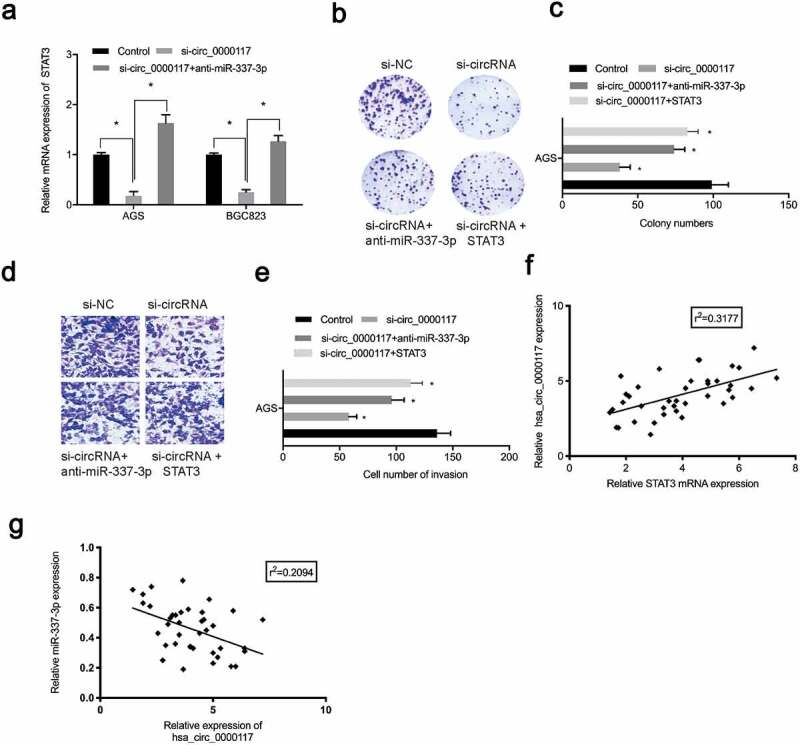
The hsa_circ_0000117/miR-337-3p/STAT3 axis promotes GC progression. (a) STAT3 mRNA expression in GC cells treated with si-circ_0000117 or si-circ_0000117 + anti-miR-337-3p. (b–e) STAT3 overexpression (or anti-miR-337-3p) abolished proliferation repression, and AGS cell invasion induced by hsa_circ_0000117 suppression. (f) The association between hsa_circ_0000117 and STAT3 in GC tissue. (g) The association between hsa_circ_0000117 and miR-337-3p in GC tissue. *P < 0.05

**Figure 7.**
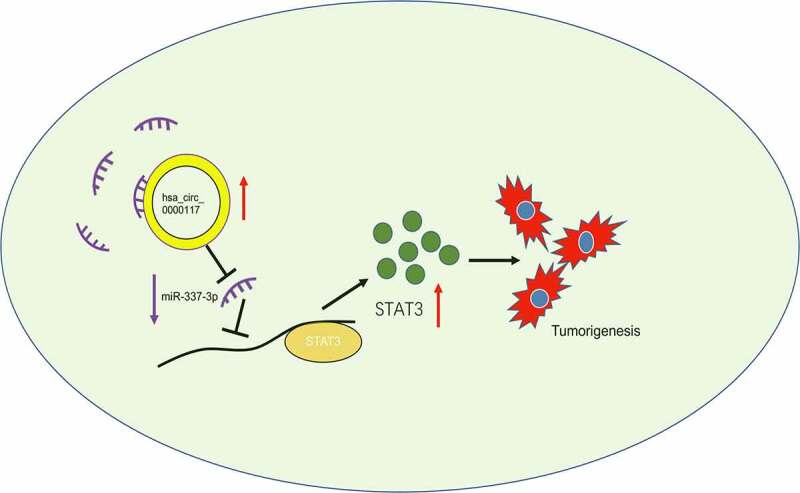
The hsa_circ_0000117/miR-337-3p/STAT3 axis in GC

To verify the role of the hsa_circ_0000117/miR-337-3p/STAT3 axis in GC progression, rescue studies were performed. Our expression data showed hsa_circ_0000117 silencing repressed STAT3, whereas anti-miR-337-3p co-transfection abolished this effect in AGS and BGC823 cells (Figure 6(a)). Our rescue assay data indicated that forced overexpression of STAT3 (or anti-miR-337-3p) partially abolished GC proliferation repression and invasion induced by hsa_circ_0000117 suppression (Figure 6(b–e)). Also, correlation analysis showed hsa_circ_0000117 expression was positively correlated with STAT3 levels, but negatively correlated with miR-337-3p levels in GC tissue (Figure 6(f–g)). Thus, hsa_circ_0000117 may function as a tumor promoter during GC progression by regulating the miR-337-3p/STAT3 axis (Figure 7).

## Discussion

Recent evidence has suggested circRNAs have crucial roles in GC tumor occurrence and advancement [19,20]. CircRBMS3 downregulation suppressed GC cell proliferation and invasion by sponging miR-153 and regulating SNAI1 expression [21]. Similarly, circNRIP1 acted as a miR-149-5p sponge to promote GC progression via the AKT1/mTOR pathway [22]. However, hsa_circ_0000117 function in GC remains unclear. In this study, hsa_circ_0000117 was increased in GC tissue and associated with advanced clinical features, consistent with previous data [12]. We also showed hsa_circ_0000117 suppression significantly decreased *in vitro* GC cell proliferation and invasion, suggesting hsa_circ_0000117 is an important regulator in GC etiology.

MiR-337-3p is a tumor inhibitor in several human cancers; it reduced ovarian cancer cell growth by regulating PIK3CA and PIK3CB [23]. Simialrly, miR-337-3p inhibited liver cancer cell progression by targeting JAK2 [24]. Moreover, loss of miR-337-3p expression in GC correlated with lymph node metastasis [16]. Consistent with these data, our results similarly provided a potential tumor-suppressing role for miR-337-3p in GC. Recent studies reported circRNAs functioned as miRNA sponges to modulate several GC biological processes. We confirmed hsa_circ_0000117 was a sponge for miR-337-3p. Equally, miR-337-3p silencing reversed hsa_circ_0000117 silencing-mediated effects on GC cell malignancy, indicating hsa_circ_0000117 regulated GC characteristics by sponging miR-337-3p.

Signal transducer and activator of transcription (STAT) proteins, i.e., STAT3, are potential targets for cancer therapy [25]. Recently, several reports showed STAT3 had vital roles in tumor progression; e.g., it was reported miR-519a enhanced chemosensitivity and promoted autophagy in glioblastoma by targeting the STAT3/Bcl2 signaling pathway [26]. Also, miR-218 served as a tumor suppressor in lung cancer by affecting the interleukin-6/STAT3 axis [27]. Similarly, the long non-coding RNA NEAT1/miR-361/STAT3 axis drove aggressive endometrial cancer progression [28]. In our research, STAT3 was highly expressed in GC tissue and associated with poor prognosis of patients, it acted as a target for miR-337-3p in these cells, and was positively regulated by hsa_circ_0000117. Thus, hsa_circ_0000117 regulated GC progression by regulating the miR-337-3p/STAT3 axis.

## Conclusion

Hsa_circ_0000117 was highly abundant in GC cells and manifested its oncogene functions by sponging miR-337-3p and increasing STAT3 expression. Hence, hsa_circ_0000117 may function as a therapeutic treatment target for GC.

## Highlight

- hsa_circ_0000117 is upregulated in GC tissues and cell line.
- hsa_circ_0000117 promotes the proliferation and invasion of GC cells.
- hsa_circ_0000117/miR-337-3p/STAT3 axis promotes GC progression.
